# A study of the dopamine transporter using the TRACT assay, a novel in vitro tool for solute carrier drug discovery

**DOI:** 10.1038/s41598-020-79218-w

**Published:** 2021-01-14

**Authors:** Hubert J. Sijben, Julie J. E. van den Berg, Jeremy D. Broekhuis, Adriaan P. IJzerman, Laura H. Heitman

**Affiliations:** 1 Division of Drug Discovery and Safety, LACDR, Leiden University, P.O. Box 9502, 2300RA Leiden, The Netherlands; 2 Oncode Institute, Leiden, The Netherlands

**Keywords:** Receptor pharmacology, Transporters in the nervous system, G protein-coupled receptors, Sensors and probes

## Abstract

Members of the solute carrier (SLC) transporter protein family are increasingly recognized as therapeutic drug targets. The majority of drug screening assays for SLCs are based on the uptake of radiolabeled or fluorescent substrates. Thus, these approaches often have limitations that compromise on throughput or the physiological environment of the SLC. In this study, we report a novel application of an impedance-based biosensor, xCELLigence, to investigate dopamine transporter (DAT) activity via substrate-induced activation of G protein-coupled receptors (GPCRs). The resulting assay, which is coined the ‘transporter activity through receptor activation’ (TRACT) assay, is based on the hypothesis that DAT-mediated removal of extracellular dopamine directly affects the ability of dopamine to activate cognate membrane-bound GPCRs. In two human cell lines with heterologous DAT expression, dopamine-induced GPCR signaling was attenuated. Pharmacological inhibition or the absence of DAT restored the apparent potency of dopamine for GPCR activation. The inhibitory potencies for DAT inhibitors GBR12909 (pIC_50_ = 6.2, 6.6) and cocaine (pIC_50_ = 6.3) were in line with values from reported orthogonal transport assays. Conclusively, this study demonstrates the novel use of label-free whole-cell biosensors to investigate DAT activity using GPCR activation as a readout. This holds promise for other SLCs that share their substrate with a GPCR.

## Introduction

Solute carrier (SLC) transporters are a large superfamily of membrane-spanning proteins that facilitate passive or secondary active transport of a wide variety of physiological and pharmacological solutes. As such, SLCs constitute important regulators of a cell’s intra- and extracellular environment and signal transduction^1^. Increasingly, the role of SLCs in onset and progression of disease is recognized^2^. This is underlined by the 21 SLCs currently targeted by clinically approved drugs and at least 10 other SLCs that have compounds in clinical trials^3^. In addition, several SLCs are known to mediate drug-drug interactions and as a result are routinely assessed in drug discovery programs^4,5^. Despite the ubiquity of these proteins in physiology and pathology only a fraction of all SLCs have been extensively investigated. This necessitates the development of methods, pharmacological tools and reagents to uncover the therapeutic potential of this relatively understudied class of proteins^6^.

One of the main challenges in propelling SLC drug discovery is the sparse implementation of high-throughput screening (HTS) strategies for in vitro functional assays^3^. Conventional transport inhibition assays based on radiolabeled or fluorescent substrate uptake^7^ pose limitations due to challenges regarding substrate synthesis, real-time measurements, and radiometric safety precautions^3^. However, cell-based assays using fluorescent membrane potential^8^, pH-sensitive or calcium-sensing dyes, can achieve impressive throughput using platforms such as fluorescent imaging plate readers (FLIPR)^9^, but may result in non-specific signals and require thorough signal validation. Another approach based on electrophysiological measurements (SURFE^2^R) can attain increased screening capacity^10^, although these assays require electrogenicity of the SLC, valid for a minority only, and often use liposome or membrane preparations. Taken together, this warrants development of novel assays that circumvent the drawbacks commonly associated with label-based or cell-free screening assays.

Label-free cell-based biosensors have been used to study a wide variety of therapeutic membrane-bound proteins including G protein-coupled receptors (GPCRs)^11,12^, receptor tyrosine kinases^13^ and ion channels^14,15^. Optical and impedance-based platforms (e.g., Epic and xCELLigence, respectively) allow real-time monitoring of changes in cell morphology, adhesion, proliferation and migration without the use of invasive and/or non-physiological labels^16,17^. Cell-based electrical impedance assays are already used as a complementary phenotypic technology for GPCR drug discovery^18^, being amenable to increased throughput screening up to 384 wells per plate^19^. Due to high sensitivity of these label-free methods, it is possible to detect GPCR activation in endogenous as well as heterologous expression cell lines^20,21^. Recently, a label-free whole-cell assay was reported by our research team in which the activity of the non-electrogenic equilibrative nucleoside transporter 1 (ENT1, also known as SLC29A1) was measured via activation of adenosine GPCRs, for which adenosine is a substrate and agonist, respectively^22^. In this work mammalian bone osteosarcoma (U2OS) cells with endogenous expression of both ENT1 and adenosine receptors were assessed using the xCELLigence technology.

In the current study we exploited the capability of the impedance-based biosensor xCELLigence to detect GPCR activation further. We developed a label-free whole-cell assay, coined the ‘transporter activity through receptor activation’ (TRACT) assay, to detect activity of the dopamine transporter (DAT, also known as SLC6A3). Here, two human cell lines with heterologous expression of DAT were used to measure DAT function via activation of an endogenous GPCR by the main substrate of DAT, dopamine. DAT, a Na^+^/Cl^–^-dependent monoamine transporter^23^, is a clinical target for treatment of attention-deficit/hyperactivity disorder^24^, narcolepsy^25^ and potentially stimulant abuse^26^, but is also an important effector in the addictive effects of psychoactive substances such as cocaine and amphetamine^27^. Due to a wealth of reported literature and availability of tool compounds, DAT was selected as a model transporter to develop this TRACT assay. The current observations demonstrate a proof-of-principle that real-time impedance measurements are suitable for the detection of dopamine-induced GPCR signaling in the absence or presence of DAT. Essentially, this allows simultaneous detection of the functional activity of two membrane-bound proteins via a single converged impedance signal. This vastly expands the possibilities for the application of label-free biosensors in SLC and GPCR drug discovery.

## Material and methods

### Chemicals and reagents

Human bone osteosarcoma cells (U2OS) were kindly provided by Mr. Hans den Dulk (Leiden Institute of Chemistry, department of Molecular Physiology, Leiden University, the Netherlands). Jump In T-REx human embryonic kidney 293 (HEK 293) cells modified for doxycycline-inducible overexpression of the wild-type human dopamine transporter (JumpIn-DAT) were provided by CeMM (Research Center for Molecular Medicine, Medical University of Vienna, Austria). Dulbecco’s Modified Eagle’s Medium high glucose (DMEM), doxycycline hyclate, dopamine hydrochloride, (±)-propranolol hydrochloride and (+)-butaclamol hydrochloride were purchased from Sigma-Aldrich (St. Louis, MO, USA). GBR12909 dihydrochloride was purchased from Toronto Research Chemicals (North York, Canada). Cocaine hydrochloride was purchased from Duchefa Farma (Haarlem, The Netherlands), where Leiden University has been certified for its use in pharmacological experiments. SCH23390 hydrochloride and raclopride were purchased from Tocris Bioscience (Bristol, United Kingdom). Yohimbine hydrochloride and doxazosin mesylate were purchased from Santa Cruz Biotechnology (Dallas, TX, USA). Radioligands [^3^H]-2β-carbomethoxy-3β-(4-fluorophenyl)-tropane ([^3^H]WIN35,428, specific activity of 82.4 Ci/mmol) and [^3^H]-(R)-(+)-7-chloro-8-hydroxy-3-methyl-1-phenyl-2,3,4,5-tetrahydro-1H-3-benzazepine ([^3^H]SCH23390, specific activity of 83.2 Ci/mmol) were purchased from PerkinElmer (Groningen, The Netherlands). xCELLigence PET E-plates 96 (ACEA Biosciences, San Diego, CA, USA) were purchased from Bioké (Leiden, The Netherlands). All other chemicals were of analytical grade and obtained from standard commercial sources.

### Stable JumpIn-DAT cell line generation

After thawing Jump In T-REx HEK 293 (JumpIn) cells were split twice a week in growth medium containing 200 µg/ml hygromycin B and 5 µg/ml blasticidin. A codon optimized ORF (Addgene #132160) for the human dopamine transporter (SLC6A3, ORF: NM_001044) was cloned into a Gateway-compatible expression vector which was generated by inserting Twin-Strep-tag epitopes followed by the human influenza hemagglutinin (HA)-tag downstream of the AttR2 gateway site in the original pJTI R4 DEST CMV TO pA vector. This vector therefore allows expression of SLC6A3 with a C-terminal Twin-Strep-HA tag. Of note, the plasmid contains two tetracycline operator 2 (TO) sites under a cytomegalovirus immediate-early (CMV) promotor to allow inducible expression of the gene of interest in the presence of doxycycline (dox). JumpIn cells were transfected with the expression vector using Lipofectamine in medium without antibiotics according to the manufacturer’s protocol (ThermoFisher Scientific). Successfully transfected cells were selected in medium containing 2 mg/ml Geneticin (G418) and 5 µg/ml blasticidin for 2–4 weeks. Resistant clones were pooled after selection and used for all further experiments.

### Cell culture

JumpIn-DAT cells were grown as adherent cells in culture medium consisting of DMEM (high glucose) supplemented with 10% (v/v) fetal calf serum (FCS), 2 mM Glutamax, 100 µg/ml streptomycin and 100 IU/ml penicillin at 37 °C and 7% CO_2_. After thawing, recovered cells were maintained up to one week in culture medium supplemented with 2 mg/ml G418 and 5 µg/ml blasticidin to select transfected clones. After this round of selection, cells were switched back to regular culture medium at least 24 h before the next experiment. Cells were subcultured twice a week at ratios of 1:8–1:16 in 10 cm plates.

U2OS cells were grown as adherent cells in culture medium consisting of DMEM (high glucose) supplemented with 10% (v/v) newborn calf serum (NCS), 2 mM Glutamax, 100 µg/ml streptomycin and 100 IU/ml penicillin at 37 °C and 7% CO_2_. Cells were subcultured twice a week at ratios of 1:8–1:12 in 10 cm plates.

### Transient U2OS-DAT cell line generation

For transient transfection of U2OS cells, empty pcDNA3.1(+) plasmid (mock cDNA), as well as cDNA encoding the human DAT (SLC6A3, ORF: NM_001044) containing a C-terminal FLAG-tag cloned into a pcDNA3.1(+) plasmid (DAT cDNA) were purchased from GenScript (Piscataway, NJ, USA). cDNA was transformed into DH5α competent cells (Invitrogen, Carlsbad, CA, USA) in the presence of 100 µg/ml ampicillin and was purified using a QIAGEN Plasmid Midi Kit (QIAGEN, Hilden, Germany). Quality and concentration of the cDNA were measured using a NanoDrop 2000 (Thermo Fisher Scientific, Waltham, MA, USA).

U2OS cells were transiently transfected using polyethyleneimine (PEI) as a transfection vector^28^. 24 h before transfection, cells were seeded in 10 cm plates to achieve 50–70% confluence on the day of transfection. Prior to transfection, medium was switched to culture medium without penicillin/streptomycin. A mix of 15 µg/ml PEI and 5 µg total cDNA (mock or DAT) in 1 ml Opti-MEM was incubated at room temperature for 30 min. Per 10 cm plate, 1 ml PEI-cDNA complex was added and cells were incubated for 24 h at 37 °C and 7% CO_2_ before membrane preparation or use in TRACT or ELISA assays.

### Whole-cell FLAG-tag ELISA

Transiently transfected U2OS cells were detached from 10 cm plates 24 h post-transfection using phosphate-buffered saline (PBS)/EDTA. Cells were counted and seeded in a sterile 96-well flat bottom plate in culture medium at 80,000 cells/well in the presence of 5 mM sodium butyrate to enhance DAT expression^29^ and incubated at 37 °C and 7% CO_2_ for 24 h (100 µl total volume). All subsequent handlings were performed at room temperature. After incubation, cells were washed with PBS and fixed with 3.7% formaldehyde for 10 min. Cells were washed with Tris-buffered saline (TBS) and subsequently blocked with TBS containing 2% (w/v) bovine serum albumin (BSA) and 0.2% (w/v) saponin for 30 min. Saponin was included in all subsequent incubation steps to facilitate membrane permeabilization^30^ to allow the primary and secondary antibodies to reach the intracellular C-terminal FLAG-tag of DAT. After blocking, cells were incubated with 1:2500 mouse anti-FLAG M2 monoclonal antibody (Sigma Aldrich) for 2 h. Subsequently, cells were incubated with 1:10,000 rabbit anti-mouse horse radish peroxidase (HRP)-conjugated IgG antibody (Sigma Aldrich) for 1 h. To visualize immunoreactivity, HRP substrate 3,3′,5,5′-tetramethylbenzidine (TMB) was added to cells and incubated for 5 min. The reaction was quenched with 1 M H_3_PO_4_. Absorbance at 450 nm was measured using a Wallac EnVision multimode plate reader (PerkinElmer, Groningen, The Netherlands).

### Whole-cell HA-tag ELISA

JumpIn-DAT cells were grown in culture medium to 80% confluence. Cells were trypsinized, counted and seeded in a sterile 96-well flat bottom plate in culture medium at 60,000 cells/well in the presence of increasing amounts (1 pg/ml–1 µg/ml) of dox (100 µl total volume). Cells were incubated at 37 °C and 7% CO_2_ for 24 h. All subsequent handlings were performed at room temperature. After incubation, cells were washed with PBS and fixed with 3.7% formaldehyde for 10 min. Cells were washed with DMEM and blocked with DMEM containing 2% (w/v) BSA and 0.2% (w/v) saponin for 1 h. After blocking, cells were incubated with 1:2500 rabbit anti-HA polyclonal antibody (Invitrogen, Carlsbad, CA, USA) for 30 min. Subsequently, cells were washed with DMEM containing 25 mM HEPES and incubated with 1:3000 goat anti-rabbit HRP-conjugated IgG antibody (Brunschwig Chemie, Amsterdam, The Netherlands) for 30 min. Immunoreactivity was visualized and measured as described in the “Whole-cell FLAG-tag ELISA” section.

### Membrane preparation

Transiently transfected U2OS-DAT cells were treated with 5 mM sodium butyrate 24 h post-transfection to enhance protein expression. U2OS-DAT cells were grown to 50–70% confluence in 10 cm plates and harvested 48 h post-transfection by scraping in PBS and pelleted by centrifugation for 5 min at 1400×g. Non-transfected U2OS cells were grown to 90% confluence prior to scraping in PBS and centrifugation. Pellets were suspended in ice-cold Tris buffer (50 mM Tris–HCl, pH 7.4) and homogenized using an Ultra Turrax homogenizer (IKA-Werke GmbH & Co.KG, Staufen, Germany). Membranes were separated from the cytosolic fraction by centrifugation at 31,000×g using an Optima LE-80K Ultracentrifuge (Beckman Coulter, Fullerton, CA, USA) for 20 min at 4 °C. Pellets were suspended in ice-cold Tris buffer, homogenized and centrifuged once more. Final pellets were suspended in ice-cold Tris buffer, aliquoted and stored at −80 °C. Protein amount of the membranes was determined using a bicinchoninic acid protein determination^31^.

### [^3^H]WIN35,428 and [^3^H]SCH23390 saturation binding assays

U2OS-DAT or non-transfected U2OS membranes (20 µg per well) were incubated in assay buffer (50 mM Tris–HCl, pH 7.4, and 100 mM NaCl) containing [^3^H]WIN35,428 or [^3^H]SCH23390, respectively, for 120 min at 25 °C to ensure equilibrium binding was reached at all tested radioligand concentrations. Total binding (TB) was determined in the presence of increasing concentrations of [^3^H]WIN35,428 (2–150 nM) or [^3^H]SCH23390 (0.1–10 nM). Non-specific binding (NSB) was determined at three concentrations of [^3^H]WIN35,428 (2, 80, 150 nM) in the presence of 100 µM GBR12909, or [^3^H]SCH23390 (0.1, 4, 10 nM) in the presence of 10 µM (+)-butaclamol. Amounts of dimethyl sulfoxide (DMSO) in all wells were kept at 1%. Membrane incubation was terminated by rapid filtration through a 96-well GF/B filter plate using a FilterMate 96-well plate harvester (PerkinElmer, Groningen, The Netherlands). Filters were washed 10 times with ice-cold assay buffer and dried completely. Filter-bound radioactivity was measured in the presence of 25 µl/well Microscint scintillation cocktail using a MicroBeta^2^ 2450 microplate scintillation counter (PerkinElmer, Groningen, The Netherlands).

### TRACT assays (xCELLigence)

Label-free whole-cell TRACT assays were performed using the xCELLigence real-time cell analyzer (RTCA) system as described in previous publications^22,32^. In principle, xCELLigence RTCA measures the impedance that is generated by cells that adhere to the gold-coated electrodes and cover the bottom of microtiter E-plates. Any change in adhesion, cell number, proliferation rate and morphology (e.g., as a result of pharmacological perturbations) is measured as an increase or decrease of impedance over time. Impedance values, which are measured continuously at a frequency of 10 kHz, for each well are converted by the RTCA software to the dimensionless parameter Cell Index (CI) using the following formula:

$$CI=\frac{({Z}_{i}-{Z}_{0})\Omega }{15\Omega }$$

where Z_i_ is the impedance at any given time point and Z_0_ is the baseline impedance that is measured at the start of each experiment^16^.

All assays were performed at 37 °C and 5% CO_2_ in 96-well E-plates in a total volume of 100 µl per well. Depending on the amount of compound additions during an experiment, background impedance at the start of each experiment was measured in 45 µl (1 addition) or 40 µl (2 additions) culture medium. Cells were seeded manually in the wells in a volume of 50 µl. Compounds were added in 5 µl per addition using a VIAFLO 96 handheld electronic 96 channel pipette (INTEGRA Biosciences, Tokyo, Japan).

#### Cell preparation and monitoring

To demonstrate reproducibility of the TRACT experiments, cells were used from at least two different cell batches. In addition, cells were used at different passage numbers, ranging from p2 to p11. U2OS-mock or U2OS-DAT cells grown to 50–70% confluence were detached 24 h post-transfection from 10 cm plates with PBS/EDTA. Background impedance in 96-well E-plates was measured using culture medium containing a final concentration of 5 mM sodium butyrate. Subsequently, cells were seeded at 40,000 cells/well in culture medium. The E-plate was left at room temperature for 30 min and placed in the recording station. Impedance was measured overnight every 15 min. Cells were treated 17–19 h after seeding based on previous reports^12,22^.

JumpIn-DAT cells grown to 70–80% confluence were briefly trypsinized from 10 cm plates prior to use in the assay. Baseline impedance was measured using culture medium containing dox (1 pg/ml–1 µg/ml) or vehicle (milliQ water). Subsequently, cells were seeded at 60,000 cells/well in culture medium. The E-plate was left at room temperature for 30 min and placed in the recording station. Impedance was measured overnight every 15 min. Cells were treated 22–24 h after seeding as dox-induced protein expression is optimal after 24 h according to the JumpIn cells manufacturer’s protocol (Thermo Fisher Scientific)^33^.

#### Cell pretreatment

In antagonist experiments, cells were pretreated by the addition of a GPCR antagonist (1 µM; SCH23390, raclopride, doxazosin, yohimbine, propranolol). In TRACT assays, cells were pretreated with a DAT inhibitor (10 µM or increasing concentrations (100 pM–10 µM); GBR12909, cocaine) or a vehicle control (0.1% DMSO in PBS). Final amounts of DMSO in each well were kept at 0.1%. Impedance was measured every minute after the addition for 60 min.

#### Cell stimulation

Cells were stimulated by the addition of dopamine (concentration depending on type of assay) or a vehicle control (1 mM ascorbic acid in PBS). Note, ascorbic acid was used in the presence of dopamine to prevent its oxidation in culture medium. In antagonist experiments, cells were stimulated with a submaximal (EC_80_) concentration of dopamine. In TRACT assays to determine the inhibitory potency of DAT inhibitors, cells were stimulated with a submaximal (EC_20_) concentration of dopamine. Impedance was measured initially every 15 s after the addition for 25 min, then every minute for 10 min, every 5 min for 50 min and finally every 15 min. For U2OS-mock and U2OS-DAT cells, impedance was measured for 120 min after stimulation. For JumpIn-DAT cells, impedance was measured for 30 min after stimulation.

### Data analysis

#### Radioligand saturation binding

To calculate *B*_max_ values, disintegrations per minute (DPM) values of each data point obtained from saturation binding experiments were converted to pmol/mg protein using the specific activity of the radioligand and overall membrane protein concentration. Resulting data were analyzed using GraphPad Prism v8.1.1 (GraphPad Software, San Diego, CA, USA). The equilibrium dissociation constant (*K*_D_) and total amount of specific binding sites (*B*_max_) of [^3^H]WIN35,438 and [^3^H]SCH23390 were determined by fitting the data with non-linear regression to an exponential one site—total and non-specific binding equation. Specific binding was visualized by subtracting linear non-specific binding from total binding and fitting the data with a one site—specific binding equation.

#### xCELLigence

Experimental data was recorded using RTCA Software v2.0 or v2.1.1 (ACEA Biosciences). CI values were normalized to the first time point prior to cell stimulation to obtain normalized CI (nCI) values. Raw nCI data were exported using RTCA Software and all subsequent analyses were performed using GraphPad Prism v8.1.1. In all experiments, nCI values of vehicle-only conditions were subtracted from all other data points to correct for vehicle-induced, ligand-independent effects. Vehicle-corrected nCI responses were analyzed by taking the absolute net area under the curve (AUC) of the first 120 min (U2OS-mock, U2OS-DAT) or 30 min (JumpIn-DAT) after agonist stimulation to make concentration-effect curves and bar graphs. Apparent potency values of dopamine (pEC_50_) and inhibitory potency values of DAT inhibitors (pIC_50_) were obtained by fitting the AUC data with non-linear regression to a sigmoidal concentration-effect curve with a pseudo-Hill slope of 1 or a variable pseudo-Hill slope.

#### Statistics

Data are shown as the mean ± standard error of the mean (SEM) of at least three separate experiments each performed in duplicate, unless stated otherwise. Significant difference between two mean potency values was determined by an unpaired two-tailed Student’s t-test. Comparison of multiple mean values to a vehicle control was done using a one-way ANOVA with Dunnett’s post-hoc test. Differences were considered statistically significant when p-values were below 0.05.

## Results

### Attenuated D_1_R-mediated dopamine response in U2OS-DAT cells

**Figure 1:**
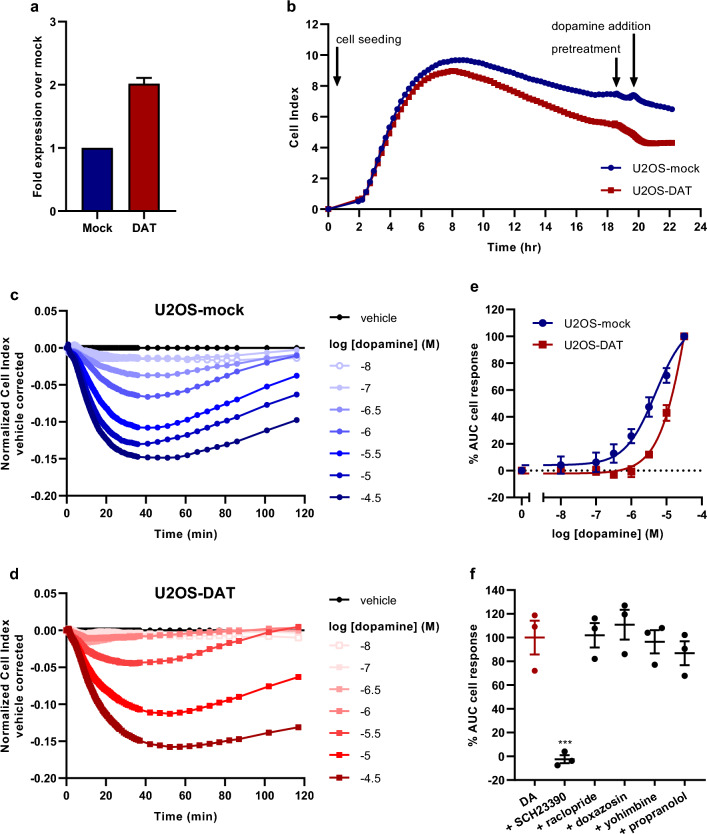
Functional characterization of dopamine (DA) response in U2OS-mock and U2OS-DAT cells in a TRACT assay. (**a**) FLAG-tag ELISA shows detection of C-terminal FLAG-tag of DAT, represented as mean ± SD of two separate experiments each performed in quintuplicate. (**b**) Representative xCELLigence growth curves after cell seeding, antagonist pretreatment and dopamine addition. (**c**) Representative vehicle-corrected xCELLigence traces of U2OS-mock and (**d**) U2OS-DAT cells after stimulation with increasing concentrations of dopamine. Data is normalized prior to agonist addition at time = 0 min. (**e**) Concentration-effect curves of dopamine on U2OS-mock and U2OS-DAT cells are shown as the net AUC of the first 120 min after stimulation normalized to the cell response of 31.6 µM dopamine. (**f**) Cell response of 10 µM dopamine (red bar, set at 100%) on U2OS-DAT cells pretreated for 1 h with 1 µM of one of following GPCR antagonists: SCH23390 (dopamine D_1_-like), raclopride (dopamine D_2_-like), doxazosin (alpha-1 adrenergic), yohimbine (alpha-2 adrenergic), propranolol (beta adrenergic). Data are shown as mean ± SEM of three to six individual experiments each performed in duplicate. Comparison of multiple mean values to vehicle control was done using a one-way ANOVA with Dunnett’s post-hoc test. *** p < 0.001.

To allow functional assessment of DAT U2OS cells were transiently transfected with DAT cDNA (U2OS-DAT) using empty vector (U2OS-mock) as a negative control. In whole-cell ELISA assays total expression of DAT was twofold higher compared to mock indicating successful transfection of U2OS cells (Fig. 1a).

To assess the responsiveness of transfected U2OS cells to dopamine in the TRACT assay U2OS-DAT and U2OS-mock cells were seeded and grown in uncoated E-plates. Cells initially adhered strongly to the wells indicated by an increase in CI between 0 and 8 h, but showed a gradual decrease in CI after 8 h (Fig. 1b). This decline in impedance likely reflects a small amount of cell death as a result of transient transfection, which was more outspoken in U2OS-DAT than in U2OS-mock. Subsequent stimulation of cells with increasing concentrations of dopamine resulted in an initial decrease in impedance that reached minimum nCI values after 40 to 60 min and then gradually returned to baseline (Fig. 1c and 1d). Both cell lines responded to dopamine in a concentration-dependent manner (Fig. 1e). Dopamine was less potent on U2OS-DAT cells (pEC_50_ = 4.0 ± 0.2) compared to U2OS-mock cells (pEC_50_ = 5.3 ± 0.2) (Table 1), showing that the presence of DAT reduces the apparent potency of dopamine.

**Table 1:** Apparent potency values of dopamine (pEC_50_) and inhibitory potency values of DAT inhibitors (pIC_50_) on U2OS-mock, U2OS-DAT or JumpIn-DAT (± dox) cells in TRACT experiments using a non-linear regression analysis model with a fixed pseudo-Hill slope of 1. Values are reported as the mean ± SEM of three to nine individual experiments performed in duplicate (*n* indicates the number of biological replicates). Significant difference between two mean potency values was determined by unpaired two-tailed Student’s t-test. *p < 0.05 (compared to U2OS-mock); ^##^p < 0.01 (compared to U2OS-DAT/dopamine); ^†††^p < 0.001 (compared to JumpIn-DAT (−dox)). Comparison of multiple mean values to vehicle control was done using a one-way ANOVA with Dunnett’s post-hoc test. ^‡‡‡^p < 0.001 (compared to JumpIn-DAT (+dox)/dopamine). ^&&&^p < 0.001 (compared to JumpIn-DAT (−dox)).

Cell line	Compound	pEC_50_ ± SEM (EC_50_ in µM)	pIC_50_ ± SEM (IC_50_ in µM)	*n*
U2OS-mock	Dopamine	5.3 ± 0.2 (5)	–	3
U2OS-DAT	Dopamine	4.0 ± 0.2* (96)	–	6
	Dopamine + 10 µM GBR12909	5.2 ± 0.2^##^ (6)	–	4
	GBR12909 + 3.16 µM dopamine	–	6.2 ± 0.1 (0.6)	4
JumpIn-DAT –dox	Dopamine	5.1 ± 0.1 (8)	–	7
JumpIn-DAT +dox	Dopamine	4.3 ± 0.0^†††^ (46)	–	9
	Dopamine + 10 µM GBR12909	4.6 ± 0.2^&&&^ (26)	–	4
	Dopamine + 10 µM cocaine	4.9 ± 0.1^‡‡‡^ (13)	–	4
	GBR12909 + 10 µM dopamine	–	6.6 ± 0.1 (0.2)	4
	Cocaine + 10 µM dopamine	–	6.3 ± 0.2 (0.6)	4

To confirm the observed dopamine response was the result of GPCR activation U2OS-DAT cells were pretreated for 1 h with 1 µM antagonist for dopamine, alpha- or beta-adrenergic receptors prior to stimulation with a submaximal concentration (10 µM) of dopamine. Pretreatment with any of the antagonists had no substantial effect on the nCI compared to cells pretreated with vehicle (Supplementary Fig. S1a). Only SCH23390, a dopamine D1-like receptor antagonist, was able to completely abolish the dopamine-induced cell response (Fig. 1f). In non-transfected U2OS cells, SCH23390, but not raclopride, eliminated the dopamine-induced response (Supplementary Fig. S1b). This demonstrates that dopamine acts as an agonist and selectively activates D_1_R endogenously expressed on U2OS cells (Fig. 1f).

### DAT inhibition with GBR12909 restores the apparent potency of dopamine in U2OS-DAT cells

**Figure 2:**
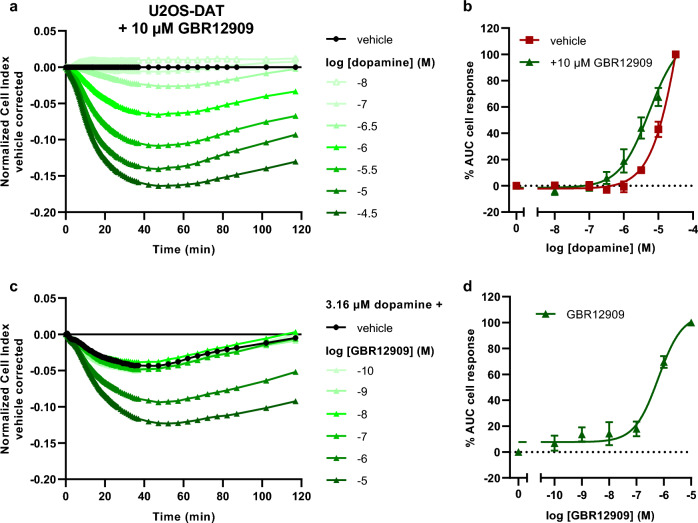
Functional characterization of GBR12909 on DAT in U2OS-DAT cells in a TRACT assay. Cells were pretreated with vehicle, 10 µM (**a,b**) or increasing concentrations (**c,d**) of GBR12909. Representative vehicle-corrected xCELLigence traces after stimulation with (**a**) increasing concentrations of dopamine or (**c**) 3.16 µM dopamine. (**b**) Concentration-effect curves of dopamine in U2OS-DAT cells pretreated with vehicle or 10 µM GBR12909 are shown as the net AUC of the first 120 min after stimulation normalized to the cell response of 31.6 µM dopamine. (**d**) Concentration-effect curve of GBR12909 after addition of 3.16 µM dopamine normalized to 10 µM GBR12909. Data are shown as mean ± SEM of three to six separate experiments each performed in duplicate.

To assess whether pharmacological inhibition of DAT leads to altered dopamine-induced D_1_R signaling in the TRACT assay U2OS-DAT cell were pretreated for 1 h with 10 µM GBR12909, an atypical DAT inhibitor, prior to stimulation with increasing concentrations of dopamine. GBR12909 pretreatment itself did not substantially affect impedance during the 1 h incubation period (Supplementary Fig. S2a). In the presence of GBR12909 dopamine induced a decrease in nCI in U2OS-DAT cells similar to that in U2OS-mock cells (compare Fig. 2a to 1c). Dopamine displayed a 16-fold higher apparent potency for D_1_R activation in U2OS-DAT cells pretreated with 10 µM GBR12909 (pEC_50_ = 5.2 ± 0.2) compared to vehicle-pretreated cells (pEC_50_ = 4.0 ± 0.2) (Fig. 2b, Table 1). Of note, the slope of the dopamine concentration-effect curve was significantly steeper (p < 0.01) in vehicle-treated cells (1.5 ± 0.1) than in cells treated with GBR12909 (0.9 ± 0.1) as was assessed by a variable slope regression model (Supplementary Table S1, Supplementary Fig. S3a). Taken together, this suggests DAT inhibition effectively prevents uptake of extracellular dopamine leading to enhanced D_1_R activation.

Next, the inhibitory potency of GBR12909 was determined in the TRACT assay. U2OS-DAT cells were pretreated for 1 h with increasing concentrations of GBR12909 and subsequently stimulated with a submaximal concentration of dopamine (3.16 µM) that resulted in the largest increase in cell response in cells pretreated with 10 µM GBR12909 (Fig. 2b). GBR12909 was able to concentration-dependently augment the dopamine-induced decrease in impedance compared to vehicle-pretreated cells with a pIC_50_ of 6.2 ± 0.1 (Fig. 2c,d, Table 1). This demonstrates that the apparent inhibitory potency of DAT inhibitor GBR12909 can be quantified in the TRACT assay.

### DAT expression is higher than D_1_R in U2OS-DAT cells

**Figure 3:**
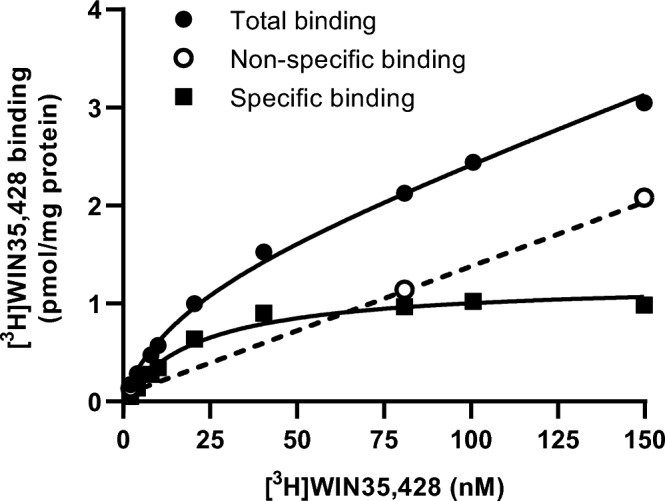
Representative saturation binding curve of [^3^H]WIN35,428 to DAT on U2OS-DAT membranes. Total binding (●) and non-specific binding (○) were determined in the absence or presence of 100 μM GBR12909. Specific binding (■) was obtained by linear subtraction of non-specific binding from total binding. Data are shown as the mean of a representative graph of three separate experiments each performed in triplicate.

To determine the relative amounts of DAT and D_1_R protein expressed on U2OS-DAT cells radioligand saturation binding experiments were performed. To this end radioligands for DAT ([^3^H]WIN35,428) and D_1_R ([^3^H]SCH23390) were used to determine the respective total amount of binding sites (*B*_max_) and equilibrium dissociation constants (*K*_D_). No specific binding of [^3^H]WIN35,428 was observed on U2OS-mock membranes (Supplementary Fig. S4a). On U2OS-DAT membranes saturable binding of [^3^H]WIN35,428 was observed with a *K*_D_ value of 28 ± 4 nM and a *B*_max_ value of 1.6 ± 0.2 pmol/mg protein (Fig. 3). The presence of D_1_R was assessed on membranes of non-transfected U2OS cells where [^3^H]SCH23390 showed saturable binding with a *K*_D_ value of 0.9 ± 0.1 nM and a *B*_max_ value of 0.1 ± 0.0 pmol/mg protein (Supplementary Fig. S4b). No detectable specific binding of [^3^H]SCH23390 on U2OS-DAT membranes was observed (Supplementary Fig. S4c). Thus, these results suggest that (at least) 16-fold more DAT is present than D_1_R on U2OS-DAT cells.

### Inducible DAT expression attenuates dopamine response in JumpIn-DAT cells

**Figure 4:**
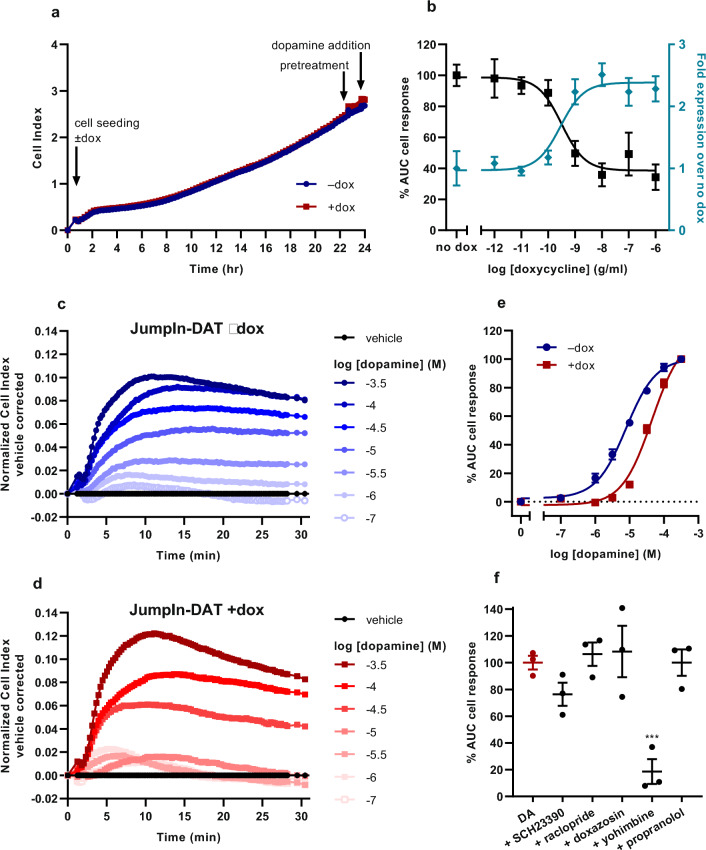
Functional characterization of dopamine (DA) response in JumpIn-DAT cells. (**a**) Representative xCELLigence growth curves after cell seeding ± 1 µg/ml dox, inhibitor pretreatment and dopamine addition. (**b**) Effect of dox on JumpIn-DAT cell response (TRACT assay) upon stimulation with 10 µM dopamine (set at 100%) (■) and effect of dox on cell surface expression of DAT detected by HA-tag ELISA (teal ♦, expressed as fold over mock). Data are shown as mean ± SD (HA-tag ELISA) or SEM (TRACT assay) of two or three separate experiments performed in quintuplicate or duplicate, respectively. (**c**) Representative vehicle-corrected xCELLigence traces of JumpIn-DAT cells in the absence of dox (–dox) and (**d**) JumpIn-DAT cells in the presence of 1 µg/ml dox (+dox) after stimulation with increasing concentrations of dopamine. Data is normalized prior to agonist addition at time = 0 min. (**e**) Concentration-effect curves of dopamine on JumpIn-DAT cells ± dox are shown as the net AUC of the first 30 min after stimulation normalized to the cell response of 316 µM dopamine. (**f**) Cell response of 31.6 µM dopamine (red bar, set at 100%) on dox-treated JumpIn-DAT cells pretreated for 1 h with 1 µM of one of following GPCR antagonists: SCH23390 (dopamine D_1_-like), raclopride (dopamine D_2_-like), doxazosin (alpha-1 adrenergic), yohimbine (alpha-2 adrenergic), propranolol (beta adrenergic). Data are shown as mean ± SEM of three to nine individual experiments each performed in duplicate. Comparison of multiple mean values to vehicle control was done using a one-way ANOVA with Dunnett’s post-hoc test. ***p < 0.001.

To validate and compare the results observed in U2OS-DAT cells a second cell line was selected. JumpIn-DAT is a HEK 293 cell line modified for dox-inducible expression of DAT. In functional label-free assays JumpIn-DAT cells were seeded and grown with or without dox in E-plates for 22–24 h. Although JumpIn-DAT cells are weakly adherent no coating was needed to detect robust CI responses. JumpIn-DAT cells attach within 4 h after seeding, which leads to a gradual increase in CI and confluence after 24 h. The presence of dox did not affect CI of JumpIn-DAT cells up to 24 h (Fig. 4a).

The amount of dox was varied to modulate levels of DAT expression. Incubating JumpIn-DAT cells for 24 h with increasing concentrations of dox enhanced cell surface expression of DAT in a concentration-dependent manner compared to vehicle-treated cells (Fig. 4b, teal diamonds). Consequently, this resulted in a dox concentration-dependent decrease in the dopamine-induced cell response (Fig. 4b, black squares), which is in line with the idea that the presence of DAT removes extracellular dopamine leading to attenuated dopamine-induced signaling. Subsequent TRACT assays with JumpIn-DAT cells were performed in the presence of 1 µg/ml dox (+dox) or vehicle (‒dox) to ensure maximal and consistent DAT expression.

To characterize the dopamine response in JumpIn-DAT cells these were stimulated with increasing concentrations of dopamine. Impedance steadily increased reaching maximum nCI values after 10 to 15 min followed by a steady plateau (−dox) or slight decrease in nCI (+dox) after 30 min (Fig. 4c,d), which is notably different from the negative nCI responses observed in U2OS cells (Fig. 1c,d). Dopamine was significantly less potent (p < 0.001) in the TRACT assay on dox-treated cells (pEC_50_ = 4.3 ± 0.0) than on vehicle-treated cells (pEC_50_ = 5.1 ± 0.1) (Fig. 4e, Table 1). This indicates that induced expression of DAT leads to extracellular removal and a decrease in the apparent potency of dopamine.

To verify dopamine-induced signaling was mediated via GPCR activation dox-treated cells were pretreated for 1 h in the presence of 1 µM GPCR antagonist prior to stimulation with a submaximal concentration (31.6 µM) dopamine. Addition of antagonists to the cells did not affect the nCI compared to cells pretreated with vehicle (Supplementary Fig. S1c). In contrast to U2OS-DAT cells the dopamine response was not affected by the dopamine D1-like receptor antagonist SCH23390, but was significantly reduced (p < 0.001) in the presence of alpha-2 adrenergic receptor antagonist yohimbine (Fig. 4f). This suggests that DAT function can be detected in JumpIn-DAT cells albeit through distinct receptor activation compared to U2OS-DAT cells.

### Characterization of DAT inhibitors is possible using JumpIn-DAT cells in a TRACT assay

**Figure 5:**
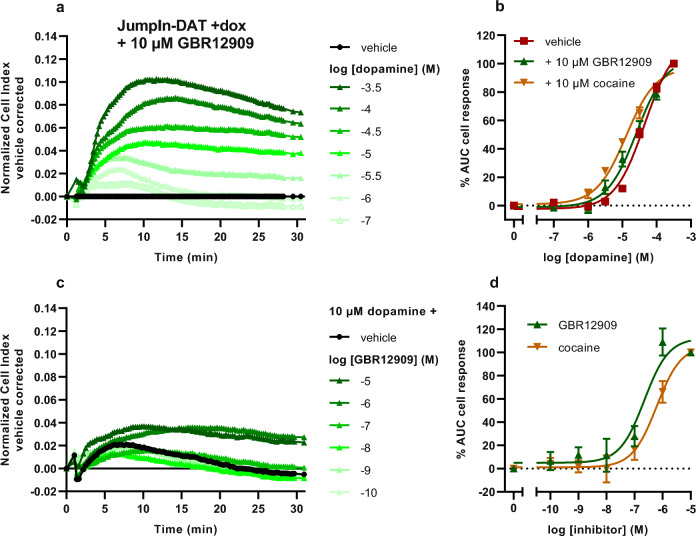
Functional characterization of GBR12909 and cocaine on DAT in JumpIn-DAT cells in the presence of 1 µg/ml dox (+dox) in a TRACT assay. Cells were pretreated with vehicle or 10 µM (**a,b**) or increasing concentrations (**c,d**) of either GBR12909 or cocaine. (**a**) Representative vehicle-corrected xCELLigence traces after stimulation with increasing concentrations of dopamine or (**c**) 3.16 µM dopamine. (**b**) Concentration-effect curves of dopamine in dox-treated JumpIn-DAT cells pretreated with vehicle or 10 µM GBR12909 or cocaine are shown as the net AUC of the first 30 min after stimulation normalized to the cell response of 316 µM dopamine. (**d**) Concentration-effect curve of GBR12909 and cocaine after addition of 10 µM dopamine normalized to 10 µM inhibitor. Data are shown as mean ± SEM of four to nine separate experiments each performed in duplicate.

After characterization of the dopamine response the inhibitory potencies of two DAT inhibitors were determined in the TRACT assay. In addition to GBR12909 the dopamine-potentiating effect of cocaine was assessed. Pretreatment of dox-treated cells for 1 h with 10 µM GBR12909 or cocaine on their own did not substantially affect impedance over time (Supplementary Fig. S2b). Subsequent stimulation with dopamine led to increased nCI values after 10 min at concentrations of dopamine between 1 and 31.6 µM compared to vehicle-pretreated cells (compare Fig. 5a to 4d). Consequently, GBR12909 enhanced the apparent potency of dopamine twofold (pEC_50_ = 4.6 ± 0.2) compared to vehicle-pretreated cells (pEC_50_ = 4.3 ± 0.0), though this was not significant (p = 0.057). Cocaine showed a significant (p < 0.001), threefold increase in apparent potency of dopamine (pEC_50_ = 4.9 ± 0.1) (Fig. 5b, Table 1). Of note, cocaine was thereby able to restore dopamine’s apparent potency to a value close to the one observed in cells without DAT (–dox: pEC_50_ = 5.1 ± 0.1; Table 1). Comparable to U2OS-DAT cells the slope of the dopamine concentration-effect curve in dox-treated cells was steeper (1.5 ± 0.1) compared to vehicle-treated cells (0.8 ± 0.1) and dox-treated cells pretreated with GBR12909 (0.8 ± 0.1) or cocaine (0.7 ± 0.1) when a variable slope regression model was used (Supplementary Table S1, Supplementary Fig. S3b).

Following characterization of DAT inhibition on JumpIn-DAT cells, the inhibitory potency of GBR12909 and cocaine was assessed on dox-treated cells. Different from U2OS-DAT cells the biggest difference in dopamine response between vehicle- and dox-treated cells was observed at a submaximal concentration (10 µM) of dopamine (Fig. 4e), proposing the optimal window to detect DAT inhibition. Both inhibitors showed concentration-dependent enhancement of dopamine-induced cell responses with GBR12909 being more potent (pIC_50_ = 6.6 ± 0.1) than cocaine (pIC_50_ = 6.3 ± 0.2) (Fig. 5c,d). This provides evidence for the suitability of the TRACT assay to determine DAT inhibitor potencies.

## Discussion

Label-free, non-invasive in vitro functional assays for SLC transporters are scarce^3^. The most prevalent strategy to assess SLC function in vitro is still by direct measurement of labeled or modified substrate uptake in recombinant cell lines, animal tissues or SLC-containing membrane preparations. Novel label-free systems that detect SLC ligand engagement in living cells, such as the cellular thermal shift assay (CETSA)^34^ and mass spectrometry binding assays^35^, require invasive processing of cells prior to or after SLC binding events. A recently described HTS-compatible label-free cell-based cytotoxicity assay for monocarboxylate transporter 1 (SLC16A1) showed promise for inhibitor screening studies^36^, although this method demands that a selective cytotoxic substrate is available for the SLC. Moreover, in 2012 Wong et al*.* reported on the use of a label-free optical biosensor to characterize functional inhibition of the electrogenic sodium-dependent phosphate transporter 2B (SLC34A2), but this was not followed up on^37^. Previously, a non-invasive assay using xCELLigence was described by our research team to detect activity of non-electrogenic ENT1 via adenosine receptor (AR) signaling in U2OS cells, which endogenously express both ENT1 and ARs^22^. Compared to the assay by Vlachodimou et al., the novelty of the current study is the use of two cell lines with distinct endogenous GPCR expression and heterologous expression of DAT. In addition, for the first time we consider the expression levels and expression ratio between the receptor and transporter, presenting a more detailed look into the mechanism of the TRACT assay and providing a guideline for its use for other SLC-GPCR pairs.

Two mammalian cell lines were used to confirm the hypothesis that the presence of DAT reduces extracellular dopamine and thereby activation of cell surface receptors. Primary criterion for cell line selection was endogenous expression of dopamine-responsive GPCRs. U2OS cells were chosen as a suitable cell line as RNA-Seq data available from The Human Protein Atlas^38^ indicated expression of D_1_R on these cells (The Human Protein Atlas: ENSG00000184845-DRD1)^39^. Moreover, functional activation of D_1_R on U2OS cells by dopamine has been reported previously in an impedance-based assay^40^. Expression of DAT is not reported in U2OS (The Human Protein Atlas: ENSG00000142319-SLC6A3^41^), which necessitated heterologous expression of DAT. Although DAT-transfected U2OS cells were successfully used to characterize pharmacological DAT inhibition (Fig. 2), the transient transfection procedure was deemed time-intensive and unfit for upscaling of experimental throughput. In addition, variation in protein expression levels and quality can vary substantially between batches of transiently transfected cells compared to stable expression systems^42^. Therefore, an additional second cell line, HEK 293 JumpIn-DAT, was created with stable and inducible expression of DAT. Reported transcriptomics data suggest that HEK 293 JumpIn cells do not express dopamine receptors (BioSamples database^43^: SAMN11893676, SAMN11893683, SAMN11893683^44–46^), but rather express the alpha-2C adrenergic receptor. Dopamine has been reported to exert agonistic effects on this receptor^47^, which was confirmed in the current study (Fig. 4f).

Uptake by DAT is the main process responsible for removal of extracellular dopamine in dopaminergic synapses and extrasynaptic spaces^48^. In striatal slices of mice dopamine released by electrical stimulation remained in the extracellular space more than 100-fold longer in DAT knock-out mice compared to wild-type mice with fully functional DAT, underlining the importance of DAT in dopamine clearance, signaling and tone^49^. Analogously, in the TRACT assay expression of DAT resulted in a lower apparent potency of dopamine compared to mock-transfected or non-induced cells assuming a pseudo-Hill slope of 1 (Figs. 1e and 5e). Interestingly, when these data were fitted to sigmoidal concentration-effect curves with a variable slope, it was evident that slopes for dopamine concentration-effect curves on U2OS-DAT and dox-treated JumpIn-DAT cells were significantly steeper compared to cells lacking DAT (Supplementary Fig. S3, Supplementary Table S1). Pretreatment with GBR12909 or cocaine restored the slopes of the dopamine concentration-effect curves in U2OS-DAT and dox-treated JumpIn-DAT cells to values close to mock or vehicle-treated cells. This observation could be explained according to concepts described by Kenakin, which postulate that a saturable removal process (e.g., dopamine uptake by DAT), of which the magnitude is dependent on the capacity of the process (V_max_) and the affinity of the substrate for the process (K_m_), affects the free concentration of a substrate present in the medium^50,51^. Thus, if the removal process is saturated within the concentration range of substrate used in the experiment, the presence of the removal process leads to an increased pseudo-Hill slope and a rightward shift of the substrate concentration-effect curve upon binding to surface receptors (e.g. GPCRs). This is the case for the TRACT assay in the current study, as dopamine K_m_ values for DAT have been reported to be between 0.1 and 5 µM in heterologous DAT expression systems^52^, which are in the range of the tested dopamine concentrations. Therefore, in this context increased pseudo-Hill slopes in addition to a rightward shift of the substrate concentration-effect curve may be indicative of a functional substrate removal process and validate the functionality of the TRACT assay.

One of the main differences between the current TRACT assay and the previously reported label-free assay for ENT1^22^ is the use of heterologous expression of the SLC. Thus, a major benefit of this approach is being able to better control the amount of SLC and/or GPCR in the cell line, making the assay less dependent on endogenous expression levels of both proteins. In this context we determined the ratio of SLC and GPCR present on the cell surface as an indication to adequately measure SLC function, by performing saturation binding experiments on U2OS-DAT cells with radioligands for both DAT and D_1_R (Fig. 3, Supplementary Fig. S2). The amount of D_1_R on non-transfected U2OS cells (*B*_max_ = 0.1 ± 0.0 pmol/mg protein) was approximately 16-fold lower than the amount of DAT on U2OS-DAT cells (*B*_max_ = 1.6 ± 0.2 pmol/mg protein). Of note, the amount of D_1_R on U2OS-DAT cells was below the detection limit of the radioligand binding assay (Supplementary Fig. S4c), indicating that the transient transfection procedure negatively impacts the expression of D_1_R on U2OS cells. It has been reported that off-target effects and changes in cell behavior upon transient transfection can be attributed to transfection reagents itself or the introduction of foreign DNA into cells^53^, which could explain the apparent reduction in detectable D_1_R in U2OS-DAT cells. This suggests that there is at least 16-fold more DAT than D_1_R in U2OS-DAT cells, which in more general terms could indicate that the SLC should be present in higher concentrations than the GPCR. This was most probably also the case in the study by Vlachodimou et al., where endogenous ENT1 is abundantly expressed on U2OS cells (B_max_ = 31 pmol/mg protein), although no saturation or expression data of adenosine receptors was reported on these cells^32^. Consequently, the transport capacity (V_max_) of the transporter to remove enough exogenous/extracellular substrate is observed as a shift in apparent substrate potency or change in pseudo-Hill slope of the GPCR response^51^. The observed differences in the apparent dopamine potency shifts between U2OS-DAT (19-fold compared to U2OS-mock) and dox-treated JumpIn-DAT (fivefold compared to vehicle-treated) may be due to differences in the method of transfection, post-translational modifications, cell surface expression levels of both DAT and GPCR, or divergent expression patterns of regulatory proteins of DAT^54^.

To validate the TRACT assay for DAT, we selected two reference DAT inhibitors (GBR12909 and cocaine) which have a 10 to 100-fold difference in affinity for DAT^55^. Both cell lines were successfully used to determine the inhibitory potency of GBR12909, which is a well-known atypical DAT inhibitor with reported low nanomolar affinity for DAT^55,56^. The presence of GBR12909 in U2OS-DAT or dox-treated JumpIn-DAT cells enhanced the response of these cells to dopamine comparable to cells lacking DAT. The inhibitory potency values for GBR12909 obtained in this study are in line with reported pIC_50_ values for a fluorescence-based neurotransmitter uptake assay (6.7)^57^ and 1-methyl-4-phenylpyridinium (MPP+)-induced toxicity inhibition (7.0)^58^, but are slightly lower compared to pIC_50_ values measured in [^3^H]dopamine uptake experiments, which show a wide range of values from 6.6 to 9.0^55,56,59–61^. This could be due to the method used to analyze the Cell Index traces of the dopamine responses (e.g., use peak nCI instead of AUC, or use different time intervals to infer the AUC), which could in turn influence the pIC_50_ value^62^. Moreover, the difference in potency may be explained by the presence of high (competing) concentrations of dopamine upon stimulation in the TRACT assay (3.16–10 µM), whereas traditional uptake inhibition assays are usually performed in the presence of 10–100 nM [^3^H]dopamine^63^. The high concentrations of dopamine could potentially mask the high affinity of compounds for DAT, as we have observed for GBR12909, which could lead to the TRACT assay detecting only compounds with a high affinity while missing out on inhibitors with low potency. However, the inhibitory potency values obtained for cocaine, a classical inhibitor that binds to the same central binding site as dopamine^64^, were in line with previously reported values measured in neurotransmitter uptake assays (pIC_50_ values range from 6.1 to 7.2)^55,56,59,60,65^, indicating that the TRACT assay is sensitive enough to detect DAT inhibitors with varying affinities.

In summary, this study reports the first label-free whole-cell bioassay, termed the TRACT assay, that allows characterization of pharmacological DAT inhibition using the impedance-based xCELLigence technology. Dopamine responses were recorded in real-time in two mammalian cell lines, each via activation of endogenously expressed GPCRs. The presence of DAT in these cells resulted in attenuated dopamine-induced GPCR signaling, which was essentially recovered upon pretreatment with DAT inhibitors. This provided an assay window to measure inhibitory potencies of two DAT inhibitors, which were in accordance with values from previously reported orthogonal functional assays. The current study demonstrates the versatility of impedance-based biosensors to detect signaling events in a single cell line, which can be attributed to both GPCR activation and SLC activity. This adds SLCs to the increasing list of protein classes that can be assessed using label-free whole cell bioassays with the intended application in drug discovery programs. Consequently, this opens up unexplored venues for development of the TRACT assay as a novel drug discovery tool for SLCs that have a shared substrate with GPCRs.

### Supplementary information

Supplementary Information

## Data Availability

The datasets generated during and/or analyzed during the current study are available from the corresponding author on reasonable request.
