# Supplementary material for: A study of the dopamine transporter using the TRACT assay, a novel in vitro tool for solute carrier drug discovery
Source: Sci Rep. 2021 Jan 14;11:1312. doi: 10.1038/s41598-020-79218-w (PMC7809260; doi:10.1038/s41598-020-79218-w)
Supplement: Supplementary file 1 — Supplementary Information [file 41598_2020_79218_MOESM1_ESM.pdf]

A study of the dopamine transporter using the TRACT assay, a novel in vitro tool for solute carrier drug discovery

Authors

Hubert J. Sijben[1], Julie J.E. van den Berg[1], Jeremy D. Broekhuis[1], Adriaan P. IJzerman[1] and Laura H. Heitman[1,2,*]

[1] Division of Drug Discovery and Safety, LACDR, Leiden University, The Netherlands

[2] Oncode Institute, Leiden, The Netherlands

* **Corresponding author**: Division of Drug Discovery and Safety, LACDR, Leiden University, P.O. Box 9502, 2300RA Leiden, The Netherlands. Tel.: +31 71 527 4558. Email: l.h.heitman@lacdr.leidenuniv.nl

Supplementary Material

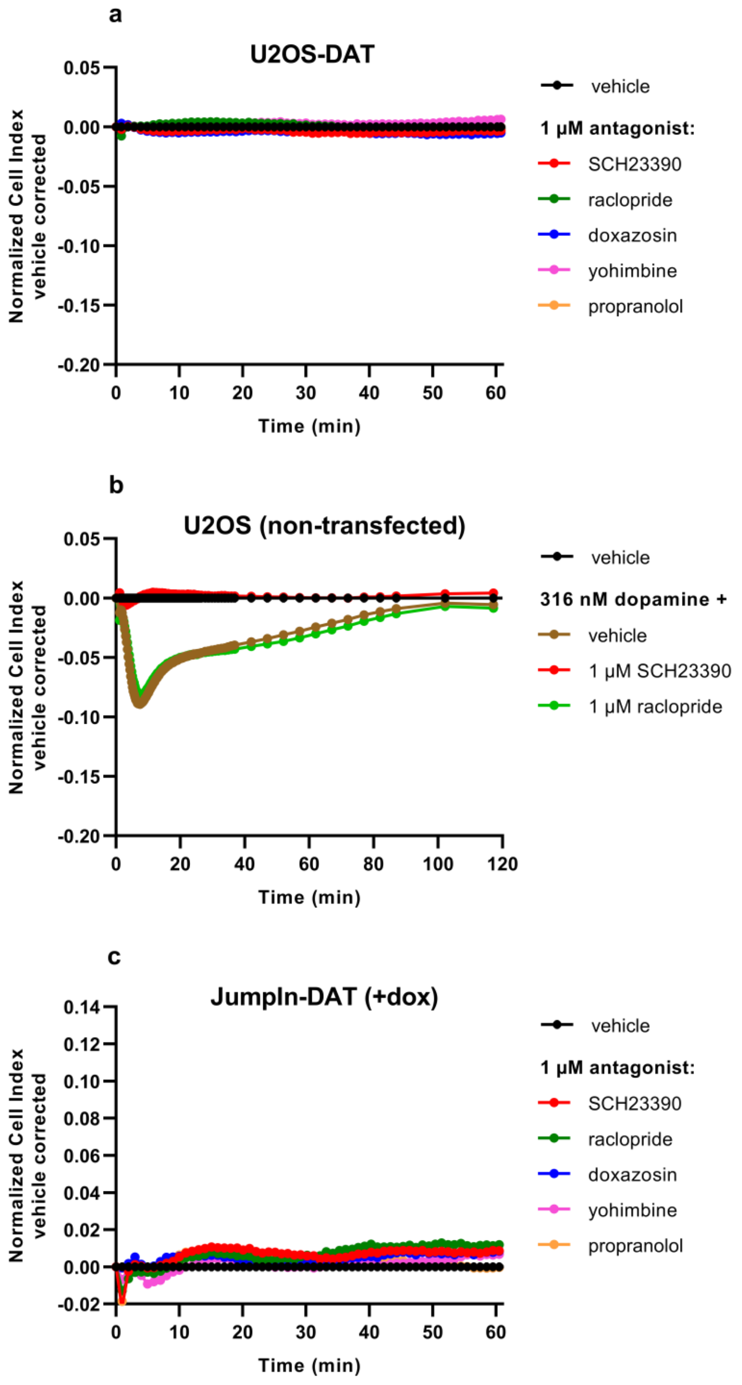

Figure S1. Representative vehicle-corrected xCELLigence traces of GPCR antagonists in three cell lines. (**a**) Pretreatment of U2OS-DAT cells with 1 μM antagonist for 1 h. (**b**) Stimulation of non-transfected U2OS cells with 316 nM dopamine after pretreatment with vehicle, 1 μM SCH23390 or raclopride for 1 h. (**c**) Pretreatment of JumpIn-DAT cells in the

presence of 1 µg/ml dox (+dox) with 1 µM antagonist for 1 h. Data are shown as the mean of a representative graph of at least three separate experiments each performed in duplicate.

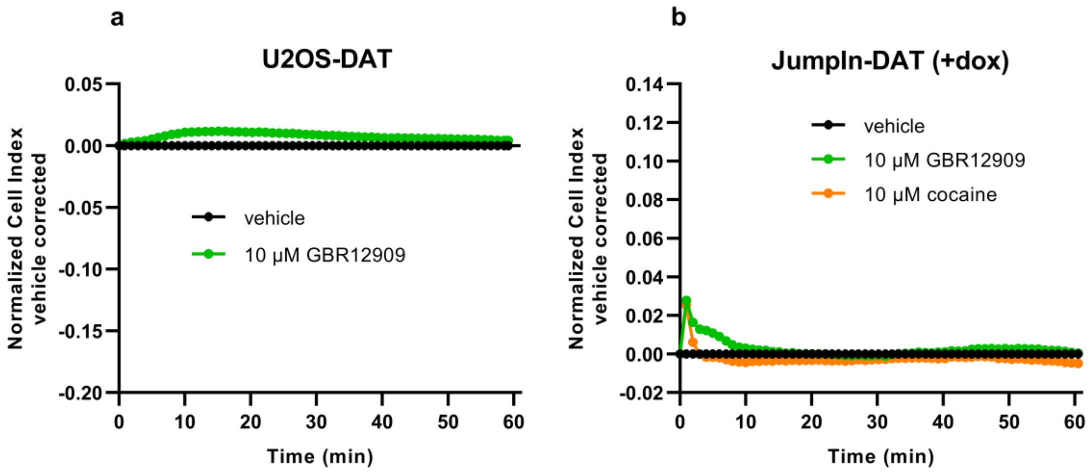

Figure S2. Representative vehicle-corrected xCELLigence traces of DAT inhibitors in two cell lines. (**a**) Pretreatment of U2OS-DAT cells with 10 µM GBR12909 for 1 h. (**b**) Pretreatment of JumpIn-DAT cells in the presence of 1 µg/ml dox (+dox) with 10 µM GBR12909 or cocaine for 1 h. Data are shown as the mean of a representative graph of at least three separate experiments performed with 16 replicates.

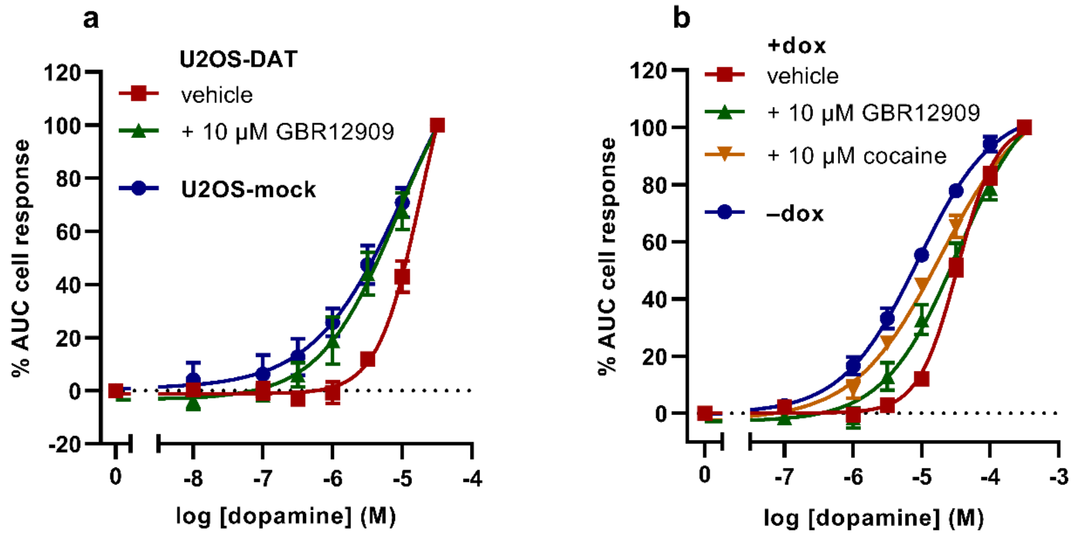

Figure S3. Dose-response curves of dopamine on (**a**) U2OS-DAT, U2OS-mock and (**b**) JumpIn-DAT cells with or without 1 µg/ml dox (±dox). Data are shown as the net AUC of the first 120 minutes (U2OS) or 30 minutes (JumpIn-DAT) after stimulation normalized to the cell response of 31.6 µM (U2OS) or 316 µM (JumpIn-DAT) dopamine. U2OS-DAT and dox-treated JumpIn-DAT cells were pretreated with 10 µM GBR12909, 10 µM cocaine (dox-treated JumpIn-DAT only) or vehicle. Data were fitted with non-linear regression to a sigmoidal dose-response curve with a variable slope. Data are shown as mean ± SEM of three to nine separate experiments each performed in duplicate.

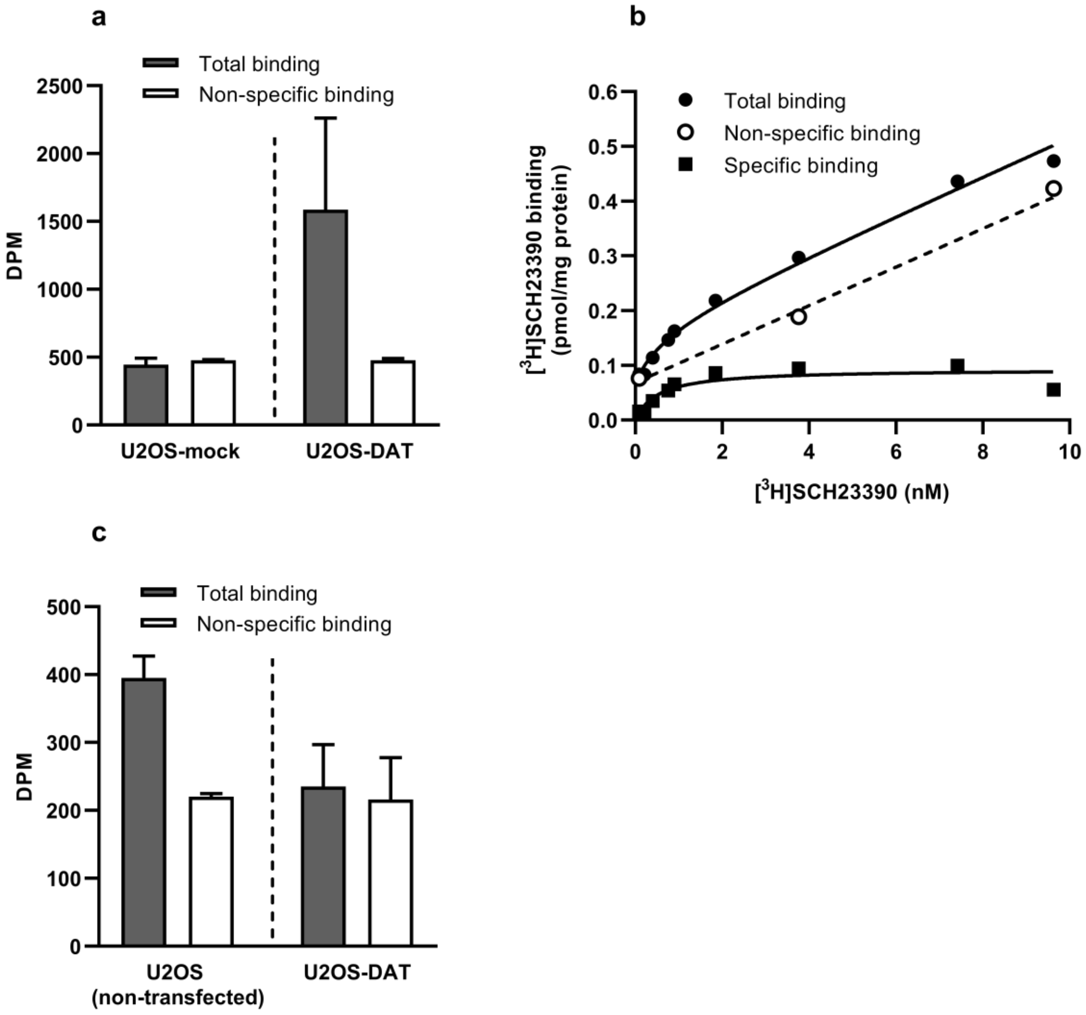

Figure S4. Radioligand binding experiments. (**a**) Binding of ~10 nM [^{3}H]WIN35,428 to 40 µg U2OS-mock or U2OS-DAT membranes. Total binding and non-specific binding were determined in the absence or presence of 100 µM GBR12909, respectively. Data are shown as mean ± SD of two experiments each performed in triplicate. (**b**) Representative saturation binding curve of [^{3}H]SCH23390 to D_1R on non-transfected U2OS membranes. Total binding (●) and non-specific binding (○) were determined in the absence or presence of 10 µM (+)-butaclamol, respectively. Specific binding (■) was obtained by linear subtraction of non-specific binding from total binding. Data is shown as the mean of a representative graph of three separate experiments each performed in triplicate. (**c**) Binding of ~0.8 nM [^{3}H]SCH23390 to 40 µg U2OS-DAT or non-transfected U2OS membranes. Total binding and non-specific binding

were determined in the absence or presence of 10 µM (+)-butaclamol. Data are shown as mean ± SD of two experiments each performed in triplicate. DPM = disintegrations per minute.

Table S1.

Apparent potency values (pEC_{50}) of dopamine and pseudo-Hill slopes of the concentration-effect curves on U2OS-mock, U2OS-DAT or JumpIn-DAT (±dox) cells in TRACT assays using a non-linear regression analysis model with a variable slope. Values are reported as the mean ± SEM of three to nine individual experiments performed in duplicate, unless stated otherwise. Significant difference between two mean potency values was determined by unpaired two-tailed Student's t-test. [†††] $p<0.001$ (compared to JumpIn-DAT (–dox)/dopamine); [‡] $p<0.05$ (compared to JumpIn-DAT (+dox)/dopamine); [##] $p<0.01$ (compared to U2OS-DAT/dopamine); *** $p<0.001$ (compared to JumpIn-DAT/dopamine). Comparison of multiple mean values to vehicle control was done using a one-way ANOVA with Dunnett's post-hoc test. [&&&] $p<0.001$ (compared to JumpIn-DAT (+dox)/dopamine).

Cell line	Compound	pEC_{50} ± SEM	slope ± SEM	*n*
U2OS-mock	Dopamine	5.4 (5.2, 5.5)[a]	0.9 (1.0, 0.8)[a]	2
U2OS-DAT	Dopamine	4.7 ± 0.2	1.5 ± 0.1	5
	Dopamine + 10 µM GBR12909	5.0 ± 0.4	0.9 ± 0.1[##]	4
JumpIn-DAT –dox	Dopamine	5.1 ± 0.1	0.8 ± 0.1	7
JumpIn-DAT +dox	Dopamine	4.5 ± 0.0[†††]	1.5 ± 0.1***	9
	Dopamine + 10 µM GBR12909	4.5 ± 0.1	0.8 ± 0.1[&&&]	4
	Dopamine + 10 µM cocaine	4.7 ± 0.1	0.7 ± 0.1[&&&]	4

[a] Mean (individual values) of two individual experiments each performed in duplicate
